# Iron Necessity: The Secret of *Wolbachia*'s Success?

**DOI:** 10.1371/journal.pntd.0003224

**Published:** 2014-10-16

**Authors:** Alessandra Christina Gill, Alistair C. Darby, Benjamin L. Makepeace

**Affiliations:** 1 Institute of Infection & Global Health, University of Liverpool, Liverpool, Merseyside, United Kingdom; 2 Institute of Integrative Biology and the Centre for Genomic Research, University of Liverpool, Liverpool, Merseyside, United Kingdom; Institut Pasteur, France

## Abstract

The bacterium *Wolbachia* (order *Rickettsiales*) is probably the world's most successful vertically-transmitted symbiont, distributed among a staggering 40% of terrestrial arthropod species. *Wolbachia* has great potential in vector control due to its ability to manipulate its hosts' reproduction and to impede the replication and dissemination of arboviruses and other pathogens within haematophagous arthropods. In addition, the unexpected presence of *Wolbachia* in filarial nematodes of medical and veterinary importance has provided an opportunity to target the adult worms of *Wuchereria bancrofti*, *Onchocerca volvulus*, and *Dirofilaria immitis* with safe drugs such as doxycycline. A striking feature of *Wolbachia* is its phenotypic plasticity between (and sometimes within) hosts, which may be underpinned by its ability to integrate itself into several key processes within eukaryotic cells: oxidative stress, autophagy, and apoptosis. Importantly, despite significant differences in the genomes of arthropod and filarial *Wolbachia* strains, these nexuses appear to lie on a continuum in different hosts. Here, we consider how iron metabolism may represent a fundamental aspect of host homeostasis that is impacted by *Wolbachia* infection, connecting disparate pathways ranging from the provision of haem and ATP to programmed cell death, aging, and the recycling of intracellular resources. Depending on how *Wolbachia* and host cells interact across networks that depend on iron, the gradient between parasitism and mutualism may shift dynamically in some systems, or alternatively, stabilise on one or the other end of the spectrum.

## Introduction

*Wolbachia*, an α-proteobacterium in the order *Rickettsiales*, has stimulated intense research interest in recent decades for four main reasons. First, it is a remarkably prevalent symbiont of invertebrates that has been described as driving a “pandemic” across terrestrial habitats, with an estimated 40% of arthropods (i.e., >1 million species) infected worldwide [1]. Second, *Wolbachia* employs a range of reproductive manipulations to drive itself by vertical transmission through arthropod populations, which could be exploited for the control of pests of medical, veterinary, or agricultural importance [2], [3]. Third, *Wolbachia* is an obligate mutualist of several filarial parasites that have a major medical or veterinary impact (e.g., *Wuchereria bancrofti*, *Onchocerca volvulus*, and *Dirofilaria immitis*), and elimination of these symbionts using antibiotics can sterilise or even kill the nematode host [4], [5]. Finally, recent research has demonstrated that *Wolbachia* can interfere with the dissemination and transmission of coinfecting microorganisms in arthropods, including arboviruses and other pathogens in mosquitoes [6]. Thus, for these latter two characteristics in particular, the biology of *Wolbachia* is highly relevant to the control of neglected tropical diseases.

**Figure 1:**
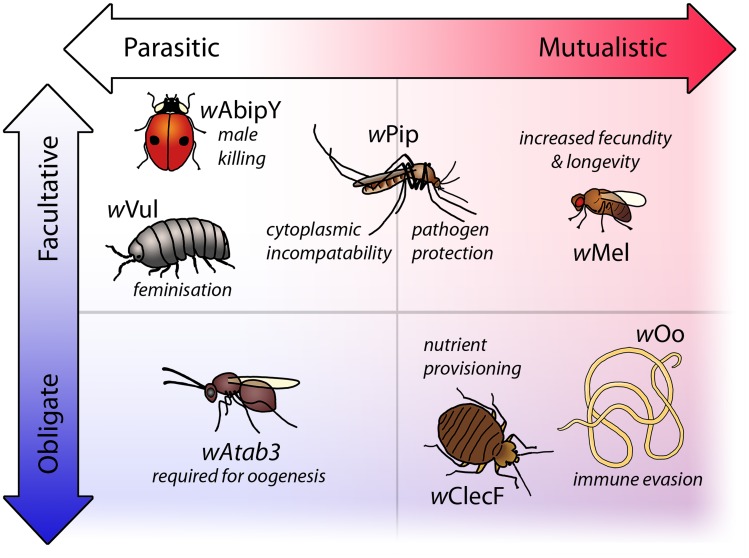
Selected examples of phenotypes resulting from natural *Wolbachia* symbioses. *Wolbachia* produces a large spectrum of phenotypes in their hosts ranging from parasitic to mutualistic traits existing as either facultative relationships or associations that have evolved to become obligate. Reproductive parasitism by *Wolbachia* is well recognised. For example, in the ladybird *Adalia bipunctata*, infection results in death of infected males during development to the benefit of female siblings (male killing) [72]; in the woodlouse *Armadillidium vulgare*, infection causes development of infected genetic males into females (feminisation) [73]; and in the mosquito *Culex pipiens*, *Wolbachia* strain *w*Pip produces cytoplasmic incompatibility (CI), in which crosses between infected males and uninfected females result in embryonic death. *Wolbachia* symbioses may also provide benefits to the host, such as increases in fecundity and longevity in *Drosophila melanogaster* [74]. In some species, mutualistic traits coexist with reproductive phenotypes, such as in *Culex pipiens*, where the CI-inducing strain *w*Pip also provides protection from mortality associated with *Plasmodium relictum* [13]. In some host species, all individuals are infected and this association is often mutualistic, as in the bedbug *Cimex lectularius* in which *Wolbachia* supplies essential B vitamins [10], or in the filarial parasite *Onchocerca ochengi*, where the presence of the bacteria is associated with the vertebrate host mounting an ineffective immune response [75]. However, in the parasitic wasp *Asobara tabida*, strain *wAtab3* is essential for oogenesis, making the relationship obligatory without any known benefits to the host [43].

The identification of endobacteria in filarial parasites as *Wolbachia* in the mid-1990s [7] had a far-reaching impact on the study of these symbionts, as clearance of the infection with antibiotics had dramatic, deleterious effects on the nematodes [4]. Importantly, obligate host–*Wolbachia* relationships had not been observed in any arthropod system at that time. However, it is now known that in a small, but diverse, selection of arthropod hosts, *Wolbachia* is essential for a wide range of reproductive processes. These include egg hatching in the collembolan *Folsomia candida* [8], oogenesis in the parasitic wasp *Asobara tabida* [9], nymphal development in the bedbug *Cimex lectularius* [10], and mate discrimination in *Drosophila paulistorum* [11]. Moreover, for both obligate and facultative *Wolbachia* symbioses, the lines between parasitism and mutualism have become increasingly blurred (Figure 1). For instance, in some populations of *Asobara japonica*, *Wolbachia* induces a type of parthenogenesis called thelytoky and has become indispensable for the production of female offspring [12]. Conversely, other populations of *A. japonica* reproduce by a second form of parthenogenesis, arrhenotoky, and do not require *Wolbachia*. In the facultative association of *Wolbachia* strain *w*Pip with the mosquito *Culex pipiens*, crosses between an infected male and an uninfected female, or a male and a female carrying different variants of *w*Pip, are rendered infertile by a phenomenon termed “cytoplasmic incompatibility.” However, despite this reproductive parasitism phenotype, the presence of *w*Pip can also benefit *C. pipiens* by reducing mortality associated with *Plasmodium relictum* infection [13]. Finally, the discovery of transfers of *Wolbachia* DNA into the nuclear genomes of nematodes that lack live, cytoplasmic infections suggests that either transient encounters with *Wolbachia* can leave their mark on the host genome, or that mutualistic relationships can break down over evolutionary timescales [14], [15].

These considerations point to a remarkable malleability in *Wolbachia*–host interactions, and it is this characteristic of *Wolbachia* that appears to set it apart from other heritable symbionts. In this review, we do not intend to examine the mechanics of vertical transmission by *Wolbachia*, nor the many hypotheses that have been developed to account for its ability to manipulate arthropod reproduction. Instead, we will survey the fundamental cellular processes that are known to be affected by *Wolbachia* infection and will focus on the intriguing interactions of this symbiont with iron-dependent pathways (Box 1), highlighting how manipulation of iron metabolism could underpin *Wolbachia*'s unmatched success in both arthropods and filariae.

**Box 1. Methods**

A literature search was performed in the Medline and Web of Science databases to identify original research articles that provided an insight into the effect of *Wolbachia* infection on iron metabolism, autophagy, apoptosis, and oxidative stress. The search term “*Wolbachia*” was used in combination with any of the following: “iron,” “autophagy,” “apoptosis,” “programmed cell death,” “oxidat*,” and “antioxidant.” In addition, recent relevant articles outside the *Wolbachia* field on haem synthesis, ferritins, and the interplay between iron, autophagy, oxidative stress, and apoptosis were identified by combining the respective search terms, but with “*Wolbachia*” omitted.

## Haem and Iron Metabolism

**Figure 2:**
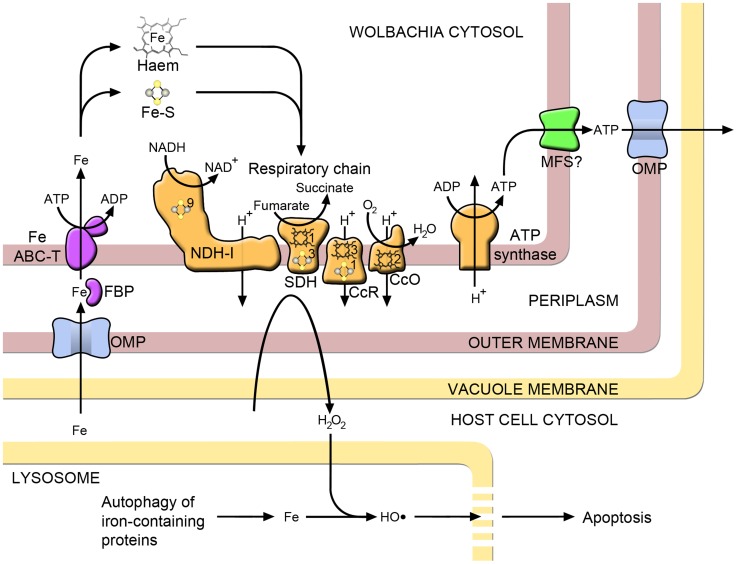
Iron metabolism and related pathways in *Wolbachia*. Iron uptake through the *Wolbachia* outer membrane may occur through a nonspecific outer membrane porin (OMP), from where it is transported across the periplasm by ferric binding protein (FBP), part of an iron ATP-binding cassette transporter system (Fe ABC-T) that moves iron into the bacterial cytosol. A major destination for iron within the bacterial cell is respiratory chain proteins, which contain iron in the form of iron–sulphur clusters (Fe-S) and haem: NADH dehydrogenase I (NDH-I), succinate dehydrogenase (SDH), cytochrome C reductase (CcR) and cytochrome C oxidase (CcO). The numbers of each of these cofactors per monomer are indicated on the relevant proteins. *Wolbachia* may export ATP generated via the electron transport chain to the host cytoplasm, possibly through a major facilitator superfamily transporter (MFS) in the inner membrane. Electron leakage from the respiratory chain generates hydrogen peroxide (H_2_O_2_). Most of this is removed by antioxidants, but some diffuses into the lysosomal compartment, where it reacts with iron to produce hydroxyl radicals. This highly reactive molecule damages the lysosomal membrane and, if sufficiently severe, apoptosis of the host cell results.

**Figure 3:**
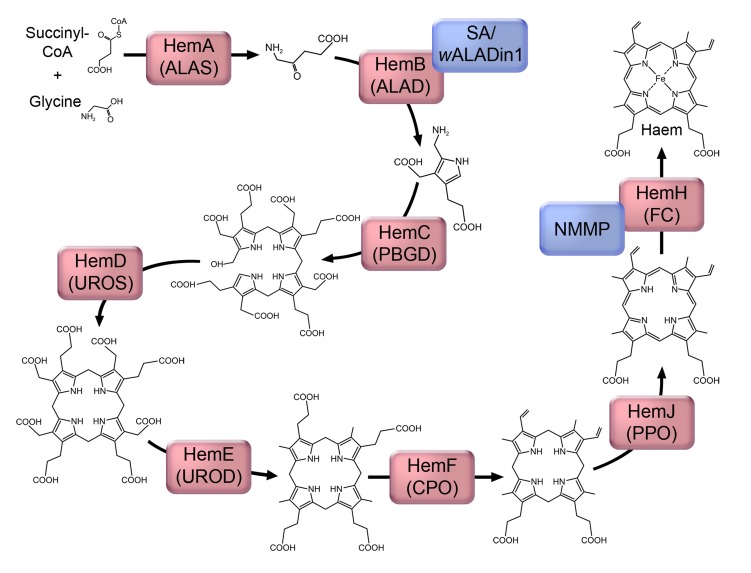
The proposed haem synthesis pathway in *Wolbachia*, showing structural intermediates. Enzymes are represented by red boxes, which contain the protein name in *Wolbachia* and the abbreviated enzyme name: ALAS, 5-aminolevulinate synthase; ALAD, 5-aminolevulinate dehydratase; PBGB, porphobilinogen deaminase; UROS, uroporphyrinogen III synthase; UROD, uroporphyrinogen III decarboxylase; CPO, coproporphyrinogen III oxidase; PPO, protoporphyrinogen IX oxidase; FC, ferrochelatase. Inhibitors of the pathway are represented by blue boxes, for which abbreviations used are as in the text.

A universal feature of all *Wolbachia* genomes sequenced to date [16]–[28] is the presence of all but one of the enzymes required to synthesise the iron-containing cofactor haem. This porphyrin is required for several essential cellular processes, including aerobic energy production, since it acts as a prosthetic group for most enzymes of the electron transport chain (Figure 2). Accordingly, an exogenous source of haem is necessary for complete development in the nematode *Caenorhabditis elegans* [29]. The missing haem synthesis gene in *Wolbachia* is protoporphyrinogen oxidase, encoded by *hemG* or *hemY* in other bacteria [30]. Although *Wolbachia* does contain genes with *hemY*-like domains [27], they are truncated by a frameshift mutation in some strains [26], and their function in haem synthesis has not been proven experimentally. However, complementation experiments have recently confirmed the existence of a further protoporphyrinogen oxidase, HemJ, in the α-proteobacterium *Rhodobacter sphaeroides* [31] and in the γ-proteobacterium *Acinetobacter baylyi* [32]. This gene has a homologue in most proteobacteria [31], including all sequenced *Wolbachia* strains, providing the missing link for de novo haem synthesis (Figure 3).

The concurrent absence of most haem synthesis enzymes in filariae gave rise to the hypothesis that *Wolbachia* has a vital role in providing this nutrient to the filarial host [16]. However, it has been shown that in adult *Onchocerca ochengi* tissues, the expression of *Wolbachia* haem biosynthesis enzymes is low, both at the protein and transcript levels. This makes an essential role for the provision of this metabolite by *Wolbachia* less likely, at least in this lifecycle stage [27]. It should also be noted that, in common with most bacteria, retention of the haem synthesis pathway is not unusual amongst the *Rickettsiales* [30]. At the same time, the pathway is universally absent from nematode genomes [33], including *Wolbachia*-negative species such as the filarial parasite *Loa loa* [33], suggesting that this species must take up haem from its hosts. It therefore seems possible that *Wolbachia*-containing filariae are also able to obtain haem from an exogenous source. There is evidence that *Litomosoides sigmodontis* [34] and *O. volvulus* [35] ingest erythrocytes from their vertebrate hosts, which would provide a rich source of haem. Further, the presence of iron-containing storage granules in the intestinal epithelium of *O. volvulus* [36] indicates the digestion of iron-containing proteins, most likely haemoglobin.

Nevertheless, an ability to take up haem would not preclude provision by *Wolbachia*, particularly at times in the lifecycle when demand is high, as is likely to be the case during moulting and embryogenesis in filarial nematodes [16]. There are precedents for provision of haem by an endosymbiont to its host: certain species of insect trypanosomatids can be cultivated in haem-deficient media if their endosymbiotic bacteria are intact. However, the addition of haem (or its immediate precursor) becomes necessary for growth when the symbionts are removed by antibiotic treatment [37]. Whether *Wolbachia* provides haem to filariae is more difficult to prove because of the obligatory nature of the symbiosis. Several studies have set out to further characterise the importance of haem with the use of inhibitors. Inhibition of the haem synthesis enzyme 5-aminolevulinate dehydratase (ALAD; Figure 3) results in adverse effects in both *Brugia malayi* [38] and *L. sigmodontis* [39]. These results would support an essential role for *Wolbachia* haem synthesis for survival of the filarial host. However, a nonspecific effect cannot be ruled out since *Wolbachia*-negative species are also affected [38], [39]. This is less marked compared with *Wolbachia*-positive nematodes, but may be explained by additional toxic effects of the ALAD substrate [39], which is likely to accumulate in these species. Disruption of haem synthesis with another inhibitor, N-methyl mesoporphyrin (NMMP) which acts on ferrochelatase (Figure 3), has also been studied. NMMP reduces the motility of *B. malayi* in vitro, even if the medium is supplemented with haem [38]. However, the interpretation of these results is not straightforward since the genome of not only *Wolbachia*, but also that of *B. malayi*, encodes a functional ferrochelatase that is inhibited by NMMP [40], [41]. Furthermore, NMMP causes a similar reduction in motility and viability of *B. malayi*, regardless of whether or not they are pretreated with antibiotics to reduce *Wolbachia* numbers [41], implying that the effect of NMMP is largely on the filarial enzyme.

Current evidence is insufficient to draw any definitive conclusions as to whether or not *Wolbachia* supplies haem to the filarial host. However, the ability to produce haem may have far wider importance in the symbiosis. In other α-proteobacteria such as *Rhizobium*, the regulation of iron metabolism is closely linked with the haem synthesis pathway [42]. Although *Wolbachia* lacks the iron response regulator (Irr) that mediates the effects of iron on gene expression in these bacteria [42], there is evidence that it is able to regulate the expression of iron-related genes in response to external stressors [43], [44]. This ability may underlie the observed fitness benefit of *Wolbachia* infection in insects reared under conditions of iron limitation [45], as well as iron overload [43], [45]. The former is likely to reflect conditions found in the wild, providing one possible mechanism for the apparent ease with which *Wolbachia* invades insect populations in nature.

## Iron and Oxidative Stress

When ferroproteins such as cytochromes are degraded by lysosomes through the process of autophagy, iron accumulates within the organelle. This iron is potentially toxic to cells, as it reacts with hydrogen peroxide to produce highly reactive hydroxyl radicals (the Fenton reaction) leading to lysosomal membrane permeabilisation and leakage of contents, which damages proteins and other cellular components (Figure 2) [46]. Cells employ a number of mechanisms to prevent oxidative damage, and *Wolbachia* infection appears to play a part.

In the parasitoid wasp *A. tabida*, iron overload disrupts development leading to reduced emergence of adults. However, this is ameliorated by *Wolbachia*, which up-regulate transcription of bacterioferritin [43]. Bacterioferritins are haemoproteins that act as iron stores and, under high iron concentrations, protect the cell from redox stress [47]. This could explain the observed protective effect in this symbiosis and provides a possible basis for the evolution of dependence of *A. tabida* on *Wolbachia*. An increase in *Wolbachia* bacterioferritin expression was also observed in *Drosophila simulans* flies fed on a high iron diet [43]. Hence, bacterioferritins may provide a means of combating iron-mediated oxidative stress in some *Wolbachia* symbioses, but not in others, as the gene is absent from several strains, including the endosymbionts of *O. ochengi* [27] and *D. immitis* [25].

There is also experimental evidence to suggest that infection with *Wolbachia* modulates redox homeostasis by altering the levels of reactive oxygen species (ROS) and affecting the expression of host antioxidants, which may have implications for host immunity [48]–[52]. However, the processes involved appear to be complex, with the net result depending on the *Wolbachia* and host strains being investigated, as well as other factors including diet. For example, in *Aedes* spp. mosquitoes, ingestion of haem leads to a decrease in ROS levels in the midgut via protein kinase signalling, which has been associated with decreased resistance to infection and increased mortality [53]. This decrease in ROS does not occur in *Aedes polynesiensis* mosquitoes infected with a native *Wolbachia* strain, suggesting manipulation of host cell signalling. However, infection with a non-native *Wolbachia* strain fails to produce this effect, implying that ROS homeostasis may be a process that has evolved over time in the natural symbiosis with *A. polynesiensis* [54]. The ingestion of an iron-rich blood meal clearly has an effect on ROS homeostasis that is altered by *Wolbachia* infection, but the underlying mechanisms remain largely unknown.

One mechanism by which *Wolbachia* is likely to contribute to oxidative stress is through generating ROS as by-products of aerobic metabolism (facilitated by high levels of lysosomal iron, Figure 2). *Wolbachia* retains key pathways for the synthesis of nucleotides [55], and their component enzymes are highly expressed by the *Wolbachia* endosymbiont (*w*Oo) in adult *O. ochengi* filariae [27]. This supports the hypothesis that a major contribution of *Wolbachia* to the filarial host could be the provision of nucleotides, and the prominence of ATP synthase in both the transcriptome and proteome of *w*Oo suggests that the most important of these is ATP generated via the electron transport chain (Figure 2) [27]. Accordingly, targeting *Wolbachia* with antibiotics results in up-regulation of components of the mitochondrial respiratory chain in *L. sigmodontis* [56]. Furthermore, doxycycline treatment of an insect cell line increased expression of elements of the *Wolbachia* electron transport chain, suggesting that the bacterium may be attempting to maintain adequate levels of energy production. In the same study, bacterioferritin and *ppnK* were up-regulated by doxycycline treatment, whereas *Wolbachia* genes involved in the assembly of iron–sulphur clusters were down-regulated, which is consistent with an attempt to limit oxidative stress [44].

## Apoptosis

Another major host cellular process that has been found to be affected profoundly by *Wolbachia* is apoptosis. Apoptosis, or programmed cell death, is an evolutionarily conserved pathway that has been shown to play an important role in normal development and survival of a wide range of multicellular organisms. Oxidative stress can lead to apoptotic cell death by initiating lysosomal membrane permeabilisation. This process is thought to be facilitated by lysosomal iron, which encourages the formation of free radicals (Figure 2) [46]. Several studies have shown an effect of *Wolbachia* on apoptosis. In *B. malayi*, depletion of the bacteria results in widespread apoptosis in the adult germ line; thus, an early effect of antibiotics on filaria is disruption of embryogenesis [57]. Similarly, in *Drosophila mauritiana*, loss of *Wolbachia* results in increased apoptosis in the germarium and reduced egg production [58], while in *A. tabida*, ovarian nurse cell apoptosis is so extensive that the endosymbiont has become indispensable for survival of the species [59]. In these insects, apoptosis is an important regulatory mechanism that is tightly controlled to allow normal oogenesis. However, the removal of *Wolbachia*, which occurs at high density in the ovaries, leads to dysregulation of this process. These observations imply that *Wolbachia* has an inhibitory effect on apoptosis. Accordingly, a major *Wolbachia* outer membrane protein inhibits neutrophil apoptosis in vitro [60], and analysis of host gene expression indicates that *Wolbachia* suppresses apoptosis in the bacteriome of a species of weevil [61]. Recent work has shown that the degree of dependence on *Wolbachia* for oogenesis in *A. tabida* varies greatly between populations and that this correlates with host ferritin expression [62], indicating that there could be a shared mechanism for interference with these host functions. Wasps that were unable to produce any eggs without *Wolbachia* showed significant increases in ferritin expression when cleared of their bacteria, while those with a lesser degree of dependence (i.e., those that were still able to produce some eggs) did not [62]. Interestingly, the effect of *Wolbachia* on apoptosis is not always inhibitory, as the pathogenic laboratory strain *w*MelPop induces apoptosis in the ovaries of *Drosophila melanogaster* [63]. It appears that the extensive apoptosis seen in the obligate mutualisms described above is caused by disruption of a complex system of crosstalk between the host and *Wolbachia*, the net result of which is influenced by coevolved host and symbiont factors.

## Autophagy

Finally, recent studies have uncovered intriguing interactions between autophagy and *Wolbachia* infection. Autophagy is the process by which cells sequester defunct or superfluous organelles and cytosolic components and deliver them to lysosomes for digestion. As such, autophagy represents an important repair mechanism that is particularly active during periods of increased cellular damage such as oxidative stress. As discussed earlier, autophagy provides a mechanism for scavenging iron from respiratory chain components, and lysosomes also provide the necessary acidic environment to liberate iron from ferritin. Moreover, oxidative damage of autophagocytosed macromolecules by intralysosomal iron leads to the production of lipofuscin, a marker of cellular aging [46]. In one study on the nematode *B. malayi*, antibodies directed at an autophagy-related protein (ATG8a) stained *Wolbachia*-rich regions. Furthermore, pharmacological triggers of autophagy resulted in loss of *Wolbachia*, while inhibition of autophagy increased bacterial numbers. Similar effects on arthropod *Wolbachia* strains were observed in *D. melanogaster* and insect cell lines [64]. Apart from having an important function in cellular homeostasis, autophagy also provides eukaryotes with a defence mechanism against pathogen invasion [65]. Accordingly, genetic inactivation of autophagy in *C. elegans* and *D. melanogaster* leads to dramatically reduced resistance to bacterial invasion [66], [67]. In light of this, the fact that *Wolbachia* levels are controlled by autophagy in diverse host species is perhaps not surprising. Like all would-be invaders, the bacteria will have had to evolve mechanisms to counteract autophagy and prevent complete removal from the host cell. In contrast to *Wolbachia*, in the closely related bacterium *Anaplasma phagocytophilum*, activation of autophagy enhances bacterial growth and inhibition of the pathway leads to reversibly arrested bacterial growth [68]. *Wolbachia*'s strategy for survival is clearly different. It is likely that the symbiont dampens the autophagic response in order to survive and replicate intracellularly, but, unlike *Anaplasma*, it does not subvert the process. Intuitively, this would seem more conducive to the infection evolving into a relatively stable symbiosis and developing mutualistic traits. This strategy makes sense considering that, in contrast to *Anaplasma*, *Wolbachia* is predominantly vertically transmitted, and its survival is therefore intricately linked to survival of the host. A study on the transcriptome of the woodlouse *Armadillidium vulgare* found that two autophagy-associated genes were down-regulated in the ovaries of symbiotic animals, which supports the theory that *Wolbachia* actively suppresses autophagy to prevent its elimination [69]. Interestingly, this mechanism appears to fail in a related species when transfected with this *Wolbachia* strain, causing catastrophic levels of autophagy that result in death of the host [70]. This suggests that the observed tolerance in *A. vulgare*, the natural host of this *Wolbachia* strain, may be due to adaptive coevolution.

## Conclusions and Future Directions

Although there are significant differences in the genomes of arthropod and filarial *Wolbachia* strains and the phenotypes that they induce in different hosts, there appear to be a remarkable number of similarities in how *Wolbachia* interacts with these hosts at the molecular level. There is evidence that *Wolbachia* is able to alter host gene expression [71] and regulate its own transcriptome in response to external factors [44], but very little is known about how it achieves this. Future efforts should be directed at attempting to gain a better understanding of these host–symbiont interactions, which may ultimately lead to advances in our ability to manipulate the symbiosis and reduce disease burden in humans and animals. This process will be facilitated by moving beyond the traditional separation of *Wolbachia* associations into mutualistic and parasitic, but considering them as variations on a theme.

The centrality of iron metabolism to energy production, oxidative stress, aging, autophagy, and apoptosis adds an additional level of complexity to the *Wolbachia*–host symbiosis that needs to be considered when interpreting the results of experiments manipulating any one of these processes. A number of priorities for future research can be identified, including: (a) the possible interaction of *Wolbachia* with lysosomes, and whether symbiont turnover provides a source of iron to host cells when this nutrient becomes limiting, (b) the role of *Wolbachia* in inhibiting or promoting cellular aging in different hosts, (c) metabolic flux analysis of aerobic respiration in infected and uninfected cells, and (d) the potential importance of iron toxicity in antibiotic-mediated killing of *Wolbachia*-infected filariae. Finally, it should be noted that many experiments investigating the role of *Wolbachia* in the symbiosis have employed antibiotics to eliminate the bacteria. However, antibiotics effective against *Wolbachia* may disrupt mitochondrial function, which also has critical roles in apoptosis, autophagy, and iron metabolism [46]. Unravelling the relationship of the host cell with *Wolbachia* from that with mitochondria is a major challenge that needs to be addressed in future studies.

**Top Five Papers**

1. Brownlie JC, Cass BN, Riegler M, Witsenburg JJ, Iturbe-Ormaetxe I, et al. (2009) Evidence for metabolic provisioning by a common invertebrate endosymbiont, *Wolbachia pipientis*, during periods of nutritional stress. PLoS Pathog 5: e1000368.
2. Kremer N, Voronin D, Charif D, Mavingui P, Mollereau B, et al. (2009) *Wolbachia* interferes with ferritin expression and iron metabolism in insects. PLoS Pathog 5: e1000630.
3. Strübing U, Lucius R, Hoerauf A, Pfarr KM (2010) Mitochondrial genes for heme-dependent respiratory chain complexes are up-regulated after depletion of *Wolbachia* from filarial nematodes. Int J Parasit 40: 1193–1202.
4. Kremer N, Dedeine F, Charif D, Finet C, Allemand R, et al. (2010) Do variable compensatory mechanisms explain the polymorphism of the dependence phenotype in the *Asobara tabida-Wolbachia* association? Evolution 64: 2969–2979.
5. Voronin D, Cook DAN, Steven A, Taylor MJ (2012) Autophagy regulates *Wolbachia* populations across diverse symbiotic associations. Proc Natl Acad Sci U S A 109: E1638–E1646.

**Key Learning Points**

- The remarkable success of *Wolbachia* as a vertically-transmitted symbiont of arthropods and its surprising distribution among filarial nematodes is unparalleled, suggesting a unique ability to integrate itself into eukaryotic physiology.
- Numerous recent studies indicate that *Wolbachia* interacts with host cell processes that are closely associated with iron metabolism. These include oxidative stress, aerobic respiration, autophagy, and apoptosis.
- The interplay between *Wolbachia* and iron metabolism occurs across the full range of host phenotypes, from reproductive parasitism (facultative) to nutrient provisioning (obligate). Thus, *Wolbachia* may use iron metabolism as a “skeleton key” to gain access to eukaryotic cellular homeostasis, leading to detrimental or beneficial host–symbiont relationships.
- Although *Wolbachia* densities are known to be controlled by autophagy, the precise nature of the interactions between the bacteria and host lysosomes are not yet understood. Lysosomes play a critical role in the recycling of iron and in the regulation of oxidative stress and aging, suggesting that they may be a key organelle in determining the phenotypic effects of the symbiosis.
- Since *Wolbachia* are obligate mutualists of many filarial nematodes and can interfere with pathogen transmission by disease vectors, dissecting the mechanisms that underpin the effects of *Wolbachia* on iron metabolism may ultimately lead to improved tools for the control of neglected tropical diseases.
